# What is the impact of the adjunctive use of omega-3 fatty acids in the treatment of periodontitis? A systematic review and meta-analysis

**DOI:** 10.1186/s12944-020-01267-x

**Published:** 2020-05-21

**Authors:** Anne B. Kruse, Carolyn D. Kowalski, Sylvia Leuthold, Kirstin Vach, Petra Ratka-Krüger, Johan P. Woelber

**Affiliations:** 1 Department of Operative Dentistry and Periodontology, Faculty of Medicine, University of Freiburg, Hugstetter Str. 55, 79106 Freiburg, Germany; 2 Private Dental Practice zahngenehm Grenzweg 28, Reinbek, DE Germany; 3 Private Dental Practice Dr. med. dent. Theres Wyss AG, Löwenstrasse 65 / Bahnhofplatz, Zürich, Switzerland; 4 Department of Medical Biometry and Medical Informatics, Faculty of Medicine, University of Freiburg, Freiburg, Germany

**Keywords:** Periodontal disease, Omega-3, Fish-oil, Eicosapentaenoic acid (EPA); docosahexaenoic acid (DHA), Host-modulation, Bleeding on probing, Pocket depth, Plaque

## Abstract

**Background:**

Host modulation therapy has gained increasing interest in periodontal therapy. This systematic review aimed to evaluate the effects of adjunctive administration of omega-3 fatty acids in periodontal therapy.

**Methods:**

The search strategy was determined using the “patient, intervention, comparison, outcome” model. A resulting search term was generated using keywords, and the databases were fed. The databases PubMed, Cochrane Library, and LIVIVO were used. Studies were selected for the literature review based on previously specified inclusion and exclusion criteria. Randomized, controlled, blinded studies, longitudinal studies, comparative studies, and clinical studies were included in the review. Additionally, they used omega-3 fatty acids in the treatment of periodontitis. The following parameters were observed: clinical attachment level (CAL), probing depth (PD), gingival index (GI), bleeding on probing (BOP) and plaque index (PI). A meta-analysis was performed for PD and CAL after 3 months. By analyzing the risk of bias, the validity of the results of each study was demonstrated, and its credibility and quality were assessed.

**Results:**

Of 14 studies found, six were included. The results showed a significant reduction in PD and CAL compared to that in the placebo groups in four out of six involved studies, which was confirmed by the meta-analysis. In one study, a significant reduction in BOP was found. GI was significantly reduced in three included studies. PI also showed a significant reduction in three studies.

**Conclusions:**

Within the study limitations, omega-3 fatty acids appear to have a positive effect on periodontal wound healing with regard to reduction in CAL and PD. Based on the results, patients receiving periodontal treatment might benefit from nutritional counseling.

## Introduction

Periodontitis is a multifactorial inflammatory disease that is increasingly responsible for tooth loss. Approximately 743 Million people are affected by severe periodontitis, making it the sixth most common disease worldwide, with a prevalence of 11.25% between 1990 and 2010 [1]. Untreated periodontal inflammation can lead to destruction of the periodontal tissue and may finally result in tooth loss. There are several influencing factors that promote the predisposition and progression of the disease, such as genetic factors, systemic diseases, and lifestyle factors including oral hygiene, smoking, stress, and nutrition [2]. Although the presence of bacterial biofilm seems crucial in the development of periodontitis, immunological host response is seen as a key factor in the disease progression [3]. Therefore, host modulation therapy is currently considered a promising treatment approach. It involves the use of local and systemic pharmaceuticals as adjuncts during periodontal therapy [4]. It aims to reduce tissue destruction by influencing inflammatory processes [5]. In theory, a number of different drugs seem suitable for use in host-modulating therapy, such as non-steroidal anti-inflammatory drugs, tetracycline, or bisphosphonates. However, they can be used only to a limited extent and have undesirable effects [6]. Within this pharmaceutical approach, polyunsaturated omega-3 fatty acids are a promising substance in the context of host-modulating therapy in numerous chronic inflammatory diseases and show fewer side effects. In this context, Chee et al. described the positive effects on periodontal inflammation in a narrative review of the literature in 2016 [7]. Omega fatty acids are considered long-chain polyunsaturated fatty acids, which are essential fatty acids because they cannot be synthesized by the body and must be ingested with food (linoleic acid, α-linolenic acid). The ratio of omega-3 to omega-6 fatty acids in tissues is largely determined by their nutritional relationship [8]. Linoleic acid is an omega-6 fatty acid that can be converted into polyunsaturated arachidonic acid (AA) in the body through dehydration and chain extension. Linoleic acid is found in high concentrations in vegetable oils, such as sunflower, safflower, soybean, and corn oils. AA is present in particularly high concentrations in foods such as meat (poultry, pork), tuna, and egg yolk. AA is also a precursor in the synthesis of prostaglandins, which act as inflammatory mediators in the body [7, 9]. The metabolism of AA occurs in two ways: lipoxins are produced by lipoxygenase, and prostaglandins are produced by cyclo-oxygenases. These derivatives have a pro-inflammatory effect. The Western diet is particularly omega-6 rich (soy, cereals, sunflower oil, and animal products) and contains few omega-3 fatty acids [10]. Omega-3 fatty acids include α-linolenic acid, eicosapentaenoic acid (EPA) and docosahexaenoic acid (DHA) in high concentrations, mainly in fish oil, as well as in rapeseed, linseed, and walnut oil [11]. Omega-3 fatty acids are incorporated into the phospholipids of cell membranes and serve as precursors for lipid mediators controlling cell signaling, gene expression, and inflammatory processes [7, 10, 11]. This leads to anti-inflammatory effects. In addition, the metabolism of omega-3 fatty acids produces so-called “pro-resolving lipid mediators”, such as resolvins and protectins with anti-inflammatory and immunoregulatory properties that control the passage of immune cells and block the production of pro-inflammatory cytokines [12, 13]. Intake of aspirin in combination with omega-3 fatty acids may result in the production of more potent resolvins and protectins, enhancing the anti-inflammatory effects [13–15]. EPA and DHA also have antibacterial properties. Thus, they may inhibit the activity of periodontal pathogens, such as *Porphyromonas gingivalis*, *Fusobacterium nucleatum*, and *Prevotella intermedia* [7, 16]*.* From the point of view of the practicing periodontist, there are still no current systematic reviews according to the authors- that have considered the effects of the adjunctive use of omega-3 fatty acids in periodontital therapy. Therefore, a general recommendation for adjunctive therapies is difficult to derive. This study aimed to filter targeted studies in a systematic review that used omega-3 fatty acids as adjunct in the treatment of periodontitis to be able to establish therapeutic recommendations.

## Material and methods

This systematic review of the literature was registered for publication at PROSPERO (https://www.crd.york.ac.uk/prospero/) under the title: “The adjunctive application of omega-3 fatty acids in the therapy of periodontitis: a systematic review “ (ID: CRD42017072234).

### Databases

The systematic literature search for the present work was performed using the following electronic databases and sources: PubMed or Medline (www.pubmed.gov)‚ Cochrane Library (www.thecochranelibrary.com), LIVIVO (www.livivo.de)‚ Science-Direct (www.sciencedirect.com).

Moreover, a hand search of leading periodontal journals was performed including the Journal of Periodontology, Journal of Clinical Periodontology, and Journal of Periodontal Research followed by a gray search.

This search was conducted until the fixed date of Octobre 8, 2019.

### Search strategies

The inclusion criteria were as follows:

- German and English language articles
- Randomized, controlled, blinded studies
- Longitudinal studies
- Comparative studies
- Clinical studies
- Use of omega-3 fatty acids in non-surgical and surgical periodontal therapy
- Recording the periodontal condition by collecting the following parameters: clinical attachment level (CAL), probing depth (PD), gingival index (GI), bleeding on probing (BOP), and plaque index (PI).

The following exclusion criteria were set:

- Abscence of periodontitis or only gingivitis
- Case reports
- Books

### Searches

A search strategy was performed using the PICO model [17], taking into account the following aspects: population/patient (patient), diagnostic/therapeutic procedure (intervention), comparison (comparison), and outcomes. Accordingly, the following search terms were defined:

Patient: periodontitis or periodontal disease

Intervention: omega 3; fish oil; EPA; DHA

Comparison: placebo

Outcomes: BOP; pocket depth; plaque.

The resulting search terms were as follows: [*(periodontitis OR periodontal disease) AND (omega 3 OR fish oil OR EPA OR DHA) AND placebo AND (bleeding on probing OR pocket depth OR plaque)].*

The selected studies were transferred to the reference and administration of a literature management program (Zotero, Roy Rosenzweig Center for History and New Media, George Mason University, Fairfax, USA), collected and saved. The studies gathered according to the search terms were systematically analyzed and sorted according to the specified exclusion and inclusion criteria.

### Meta-analysis

In the meta-analysis, all included studies were examined for mean differences and standard errors. In case of missing data, the corresponding authors were contacted via e-mail.

### Risk of bias rating

By analyzing the risk of bias, the validity of the results of each study was presented, and their credibility and quality were assessed (Cochrane Germany, [18]). A distinction was made between internal and external validity.

The following quality criteria were defined for the evaluation:

A) General bias (according to Cochrane Germany or Schmucker et al. [18])
  - Study design
  - Randomization.
  - Blinding.
  - Definition of inclusion and exclusion criteria.
  - Number of study participants.
  - Number of participants per study group.
  - Mention of dropouts.
  - Control over the compliance of participants.
  - Presentation of significant results.
B) Omega-3 study-related bias (according to Chee et al. [7])
  - Information on medication and composition.
  - Information on dose.
  - Information on the duration of the medication.
  - Indication of placebo (composition).
  - Systemic inflammation (C-reactive protein [CRP]).
C) Periodontal therapy-related bias (according to Schmidt et al. [19])
  - Classification of periodontal disease.
  - Selection of patients.
  - Treatment.
  - Periodontal parameters orindices.
  - Number of examiners.
  - Calibration of the examiner.
  - Time of collection of periodontal parameters.
  - Systemic diseases.
  - Risk factors, such as smoking.

The characteristics were categorized into three stages:

1. Quality criterion fulfilled (1 point): clear details of the criterion are provided in the available study.
2. Quality criterion not met (0 point): the criterion is mentioned, but there is no clear indication of the criterion in the relevant study.
3. Quality criterion unclear (N/A): the criterion is not mentioned.

**Grading of Recommendations Assessment, Development and Evaluation (GRADE).**

The quality of findings was assessed using GRADE according to Schünemann also stating the importance of the results [20].

## Results

### Search results

**Fig. 1:**
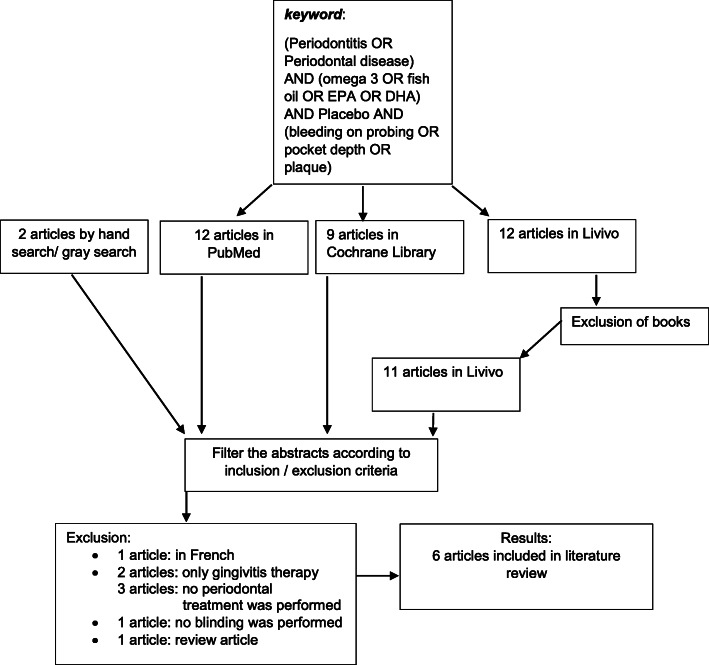
Systematic literature search in PubMed, Cochrane Library, Livivo with the defined search term and by hand search/ grey search

Through the database search, 12 articles in PubMed and 12 articles in LIVIVO were found with the specified search term. In the Cochrane Library, 9 articles were found. In LIVIVO, besides articles, some search results from books were also cited as sources, whereby the number of studies found was sometimes higher. One more article was found through hand search of periodontal journals. Furthermore, a gray search revealed another article. After filtering the specified search terms, 12 studies were included in the analysis of the selection criteria (Fig. 1).

Subsequently, the abstracts of these articles were examined for compliance with the criteria. Eight more articles were excluded after full-text analysis. The article by Lourenço et al. was excluded because it is an animal study [21]. The articles by Naqvi et al. [22, 23] and Rosenstein et al. [24] were excluded because only oral hygiene instruction was provided for the duration of the study, but no periodontal treatment was performed. The articles by Campan et al. and Jenabian et al. were excluded because only gingivitis was investigated [25, 26]. The article by Rampally et al. was excluded because of a missing specification if blinding was performed [27]. All other studies were blinded. The article by Stando and Lewkowicz was excluded because it was a review article [28]. Accordingly, five articles of the following authors were finally included in the literature review: Elwakeel and Hazaa (2015) [29], Deore et al. (2014) [30], Elkhouli (2011) [13], El-Sharkawy et al. (2010) [15], Martinez et al. (2014) [31] and Keskiner et al. (2017) [32].

### Search results - second rating

A second independent control of the search results was conducted by another rater (SL). Using the abovementioned search terms, the indicated databases were searched.

The articles found in the search between the two independent readers had a 100% match (Po = 1, Cohen’s kappa = 1.0). With a Cohen’s kappa of 1.0, the interrater variance was in an excellent range.

### Included studies

After completion of the search, six studies were included in the literature review according to the criteria (Table 1).

**Table 1:** Filtering of the studies found according to the predetermined selection criteria. Left: List of articles found by the search. Top: the specified inclusiocriteria, based on which the analysis of the articles took place. Legend: x = criterion applies, o = criterion does not apply, bold font = these studies were included in the literature review, *italic font* = these articles have been excluded from the literature review

Studies	Clinical Trial	RCT	Human	Periodontitis	Adjunctive ω3-FS	Adjunctive aspirin	Non-surgical periodontal therapy	Surgical periodontal therapy	Placebo control	Probing depth	Clinical attachment level	Gingiva index	Bleeding on probing	Plaque index	CRP
**Elwakeel et al. 2015** [29]	x	x	x	x	x	x	x	o	x	x	x	x	o	x	o
*Naqvi* et al. *2014* [22]	x	x	x	x	x	x	o	o	x	x	o	x	x	x	x
**Deore et al. 2014** [30]	x	x	x	x	x	o	x	o	x	x	x	x	x	o	x
**Martinez et al. 2014** [31]	x	x	x	x	x	o	x	o	x	x	x	o	x	x	o
**Elkhouli 2011** [13]	x	x	x	x	x	x	x	x	x	x	x	x	x	x	o
**El-Sharkawy et al. 2010** [15]	x	x	x	x	x	x	x	o	x	x	x	x	x	x	o
*Rosenstein* et al. *2003* [24]	x	x	x	x	x	o	o	o	x	x	o	x	o	x	o
*Campan* et al. *1997*[33]	x	x	x	o	o	o	o	o	x	o	o	x	o	x	o
*Campan* et al.*1996* [26]	x	x	x	o	o	o	o	o	x	o	o	x	o	x	o
*Jenabian* et al. *2012*[25]	x	x	x	o	x	o	o	o	x	o	o	x	x	o	o
*Naqvi* et al. *201 7* [23]	x	x	x	x	x	x	o	o	x	x	x	x	x	x	o
*Lourenço* et al. *2018* [21]	x	x	o	x	x	o	o	o	x	x	o	x	o	x	x
*Rampally* et al. *2019* [27]	x	x	x	x	x	x	x	o	x	x	x	x	o	x	o
**Keskiner et al.2017** [32]	x	x	x	x	x	o	x	0	x	x	x	x	x	x	o

### Elwakeel and Hazaa 2015 [29]

In a randomized, controlled trial, Elwakeel and Hazaa (2015) investigated the effect of omega-3 fatty acids (1000 mg, 3 times daily) in combination with low-dose aspirin (75 mg, once daily) in nonsurgical periodontal therapy in patients with periodontitis and type 2 diabetes. Forty patients were divided into two groups. For a period of 6 months, group 1 received omega-3 fatty acids in combination with aspirin, and group 2 received a placebo adjunctively to subgingival instrumentation. It was shown that, in group 1, PD, PI, GI, and CAL significantly decreased. The HbA1c value decreased but was not significantly different between the groups. Additionally, there was a significant reduction in the IL-1β value in the sulcus fluid in both groups.

### Deore et al. (2014) [30]

In this randomized, controlled, double-blinded clinical study, the extent to which the adjunctive administration of omega-3 fatty acids reduced clinical inflammatory parameters was examined. Two groups (60 patients) received oral hygiene instructions and non-surgical periodontitis treatment with subgingival instrumentation. Group 1 received concomitant omega-3 fatty acids (300 mg, once daily) for 12 weeks, and group 2 received a placebo. At baseline and 6 and 12 weeks, the PI, GI, oral hygiene index (by Greene and Vermillion [1964]), BOP, PD in four-point measurement, CAL, and serum CRP level were recorded. There was a significantly greater reduction in GI, BOP, PD, and CAL in group 1 compared to those in the control group. No significant changes were noted in the PI and serum CRP level.

### Elkhouli (2011) [13]

A controlled, double-blinded, clinical study examined the efficacy of systemic adjunctive administration of omega-3 fatty acids in combination with low-dose aspirin in the regenerative treatment of furcation defects. Forty patients with at least one grade 2 furcation defect were enrolled in the study and divided into two groups. First, oral hygiene instructions and subgingival instrumentation were performed. In conjunction with the use of bone substitute material for furcation coverage, one group received omega-3 fatty acids (1 g, 3 times daily) and aspirin (75 mg, once daily), and the other group received a placebo. Clinical parameters were measured at baseline and 3 and 6 months (PI, GI, gingival bleeding index, PD, CAL). Moreover, the IL-β and IL-10 levels in the sulcus fluid were determined, and a significant reduction was observed after the ingestion of omega-3 fatty acids. In the omega-3 group, there was a significant reduction in PD, PI, GI, and CAL at 6 months compared to the control group.

### El-Sharkawy et al. (2010) [15]

In this clinical study, the effect of omega-3 fatty acids in combination with low-dose aspirin on the treatment of chronic periodontitis was investigated. Eighty patients were divided into two groups. Periodontitis was treated with subgingival instrumentation, with one group receiving a concomitant omega-3 fatty acids (900 mg EPA + DHA) and aspirin (81 mg) and the other group receiving placebo. Clinical parameters at baseline and 3 and 6 months included PI, GI, BOP, PD, and attachment level. Nuclear factor kappa B ligand (RANKL) and matrix metallproteinase-8 (MMP-8) were also determined in saliva samples. There was a significant reduction in PD and CAL in the group receiving omega-3 fatty acids compared to those in the control group. The PI showed no significant difference between the control group and omega-3 group. GI was significantly reduced in both groups, but there was no significant difference between the two groups. The BOP also showed no significant difference between the two groups. Salivary RANKL and MMP-8 levels were significantly reduced in the omega-3 group.

### Martinez et al. (2014) [31]

In this randomized clinical trial, 15 patients with generalized chronic periodontitis underwent non-surgical periodontal treatment. Seven patients in the test group received omega-3 fatty acid capsules (180 mg EPA, 120 mg DHA) three times daily for 12 months. The control group (8 patients) received a placebo during this period. The periodontal parameters (PD, PI, CAL, and BOP) and serum DHA, EPA, DPA, and AA levels were controlled at baseline and at 4 and 12 months. The percentage of positive BOP sites significantly decreased in the placebo group at 4 and 12 months compared to that at baseline. There was a significant improvement in PD and CAL in both groups at 4 and 12 months, but there were no significant differences in clinical parameters between the two groups. Additionally, an increase in serum EPA levels and a reduction in the AA/EPA ratio were observed at 12 months.

### Keskiner et al. (2017) [32]

This randomized placebo-controlled clinical trial included 30 patients who received non-surgical periodontal treatment with a diagnosis of chronic periodontitis. After oral hygiene instructions and subgingival periodontal treatment 15 patients (test group) received low-dose omega-3 fatty acid capsules (DHA 19.19 mg and EPA 6.25 mg, twice daily for 6 months), while 15 patients in the control group received placebo capsules. At baseline and after 1, 3, and 6 months, the periodontal parameters (PD, CAL, BOP, GI, and PI) were examined. Additionally, saliva samples were obtained at the same time points and analyzed for tumor necrosis factor (TNF)-alpha and superoxide dismutase. The results showed significant changes in clinical parameters (reduction in PD, CAL, BOP, GI, and PI) in both groups at different time points compared to that at baseline. However, no statistically significant differences between the test and control group were found at any time point. Salivary TNF-alpha levels in the test group significantly decreased at 6 months compared to that in the control group.

### Study design

A comparison of the literature results (Table 2) showed that all studies were clinical, randomized, controlled, blinded trials. The number of patients in the investigations was between 15 and 80. The study duration was between 3 and 12 months.

**Table 2:** Comparison of study design: Left: Study parameters considered. Top: Included studies. Yes / no: criterion fulfilled / not met

	Elwakeel und Hazaa (2015) [29]	Deore et al. (2014) [30]	Elkhouli (2011) [13]	El-Sharkawy et al. (2010) [15]	Martinez et al. (2014) [31]	Keskiner et al. (2017) [32]
Clinical trial	Yes	Yes	Yes	Yes	Yes	Yes
Randomised, controlled	Yes	Yes	Yes	Yes	Yes	Yes
Chronic periodontitis (CP)	moderate-severe CP + diabetes type2	moderate-severe CP	moderate-severe periodontitis +at least 1 furcation degree 2	severe CP	light, moderate, severe CP	Yes
Gingivitis	No	No	No	No	No	No
Smoker	No	No	Yes (< 10/day)	No	Yes	No
Omega3-fatty acids dosage	1000 mg, 3x/ day for 6 months	300 mg(180 mg EPA, 120 mg DHA), 1x/day for 12 weeks	1000 mg (300 mg DHA+ 150 mg EPA) 3x/day for 6 months	900 mg EPA, 900 mg DHA, wheat germ oil	300 mg (each 180 mg EPA+ 120 mg DHA) 3x/day for 12 months	6.25 mg EPA, 19.19 mg DHA 2x/day for 6 months
Aspirin	75 mg, 1x/day for 6 months only test group	No	75 mg 1x /day für 6 months	81 mg 1x /day	No	No
Placebo	Coconut oil for omega-3, lactose for aspirin	300 mg paraffin / day	Yes	Yes	Yes	Yes
Number of patients	40 (20/20)	60 (30/30)	40 (20/20)	80 (40/40)	15 (test 7/control 8)	30 (15/15)
Number of examiners	1	1	1	at least 2 calibrated	> 1 exact specification is missing,calibrated	1
Study period	6 months	3 months	6 months	6 months	12 months	6 months
Oral hygiene instructions	Yes	Yes	Yes	Yes	No	Yes
Subgingival instrumentation	Yes	Yes	Yes	Yes	Yes	Yes
Clinical periodontal parameter		OHIS				
GI	Yes	Yes	Yes	Yes	No	Yes
PI	Yes	Yes	Yes	Yes	Yes	Yes
BOP	No	No (only at baseline)	Gingival bleeding index	Yes	Yes	Yes
PD	Yes	Yes	Yes	Yes	Yes	Yes
CAL	Yes	Yes	Yes	Yes	Yes	Yes
Recording of the parameters	0, 3, 6 months	0, 1.5, 3 months	0, 3, 6 months	0, 3, 6 months	0,4,12 months	0,1,3,6 months

### Type of periodontal disease

The studies evaluated the influence of omega-3 fatty acids on periodontitis in varying degrees of severity. Elwakeel and Hazaa (2015), Deore et al. (2014), and Elkhouli (2011) investigated patients with moderate to severe periodontitis. In the study by El-Sharkawy et al. (2010), only patients with severe periodontitis were included. In the study by Elkhouli (2011), there was an additional admission criterion that at least one tooth in the dentition had to have grade 2 furcation. Martinez et al. (2014) included patients with generalized chronic periodontitis regardless of severity. Keskiner et al. (2017) included patients with chronic periodontitis having at least nine posterior teeth with 5–7 mm PD, not including third molars and teeth with restorations such as bridges and crowns and three teeth with ≥6 mm of clinical attachment loss.

### Risk factor smoking

In the studies by Elwakeel and Hazaa (2015), Deore et al. (2014), Keskiner et al. (2017), and El-Sharkawy et al. (2010), smoking was an exclusion criterion. The studies by Elkhouli (2011) and Martinez et al. (2014) included smoking patients. However, Martinez et al. (2014) included only one smoker, who was in the placebo group. Elkhouli (2011) excluded smokers who consumed ≥10 cigarettes a day. However, it was not described if and how many smokers with < 10 cigarettes a day were included in the examinations and which group they belonged to.

### Omega-3 fatty acids, aspirin, and placebo

Analysis of the literature showed that there were differences in individual studies regarding the selection, dosage, and duration of adjuvant administration of omega-3 fatty acids. The omega-3 fatty acids, EPA, and DHA were administered in capsule form (Table 2). In the study by El-Sharkawy et al. (2010), wheat germ oil was added. Elwakeel and Hazaa (2015) did not specify the composition of the omega-3 fatty acid tablet. The dose of omega-3 fatty acids greatly varied between the studies. In the study by Deore et al. (2014), 300 mg was administered only once daily, and in comparison, the patients in the studies by Elwakeel and Hazaa (2015) and Elkhouli (2011) received 1000 mg 3 times daily. El-Sharkawy et al. (2010) administered a combined preparation containing 900 mg each of EPA and DHA. Martinez et al. (2014) administered EPA (180 mg) and DHA (120 mg) three times daily. In the study by Keskiner et al. (2017), the patients in the test group received capsules containing 6.25 mg EPA and 19.19 mg DHA from Atlantic salmon (Vectomega tablet, Laboratoires Le Stum, Plage, France). The placebo was identical, except for the fish oil‚ being prepared at the Gulhane Military Medical Academy.

There were also differences in the duration of administration. In the study by Martinez et al. (2014) patients received the dose for a longest period of 12 months. A six-month duration was observed by Elwakeel and Hazaa (2015), Elkhouli (2011), Keskiner et al. (2017), and El-Sharkawy et al. (2010). A short administration of 3 months was mentioned in the study by Deore et al. (2014). In three of five studies, aspirin was administered daily in addition to omega-3 fatty acids. The dose was either 75 mg/day (Elkhouli [2011], Elwakeel and Hazaa [2015]) or 81 mg/day (El-Sharkawy et al. [2010]). In all studies, patients in the control group received a placebo.

### Periodontal therapy

Martinez et al. (2014) only performed subgingival instrumentation without mentioning previous oral hygiene instructions. In the other five studies, patients also received oral hygiene instructions (Table 2). Elkhouli’s (2011) study also included regenerative furcation therapy.

### Outcome parameters

**Fig. 2:**
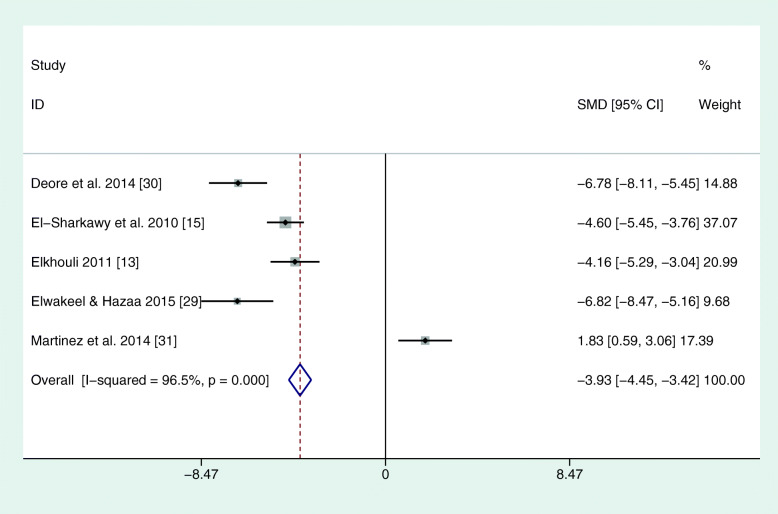
Meta-analysis of PD after 3 months. SMD = standardized mean differences

**Fig. 3:**
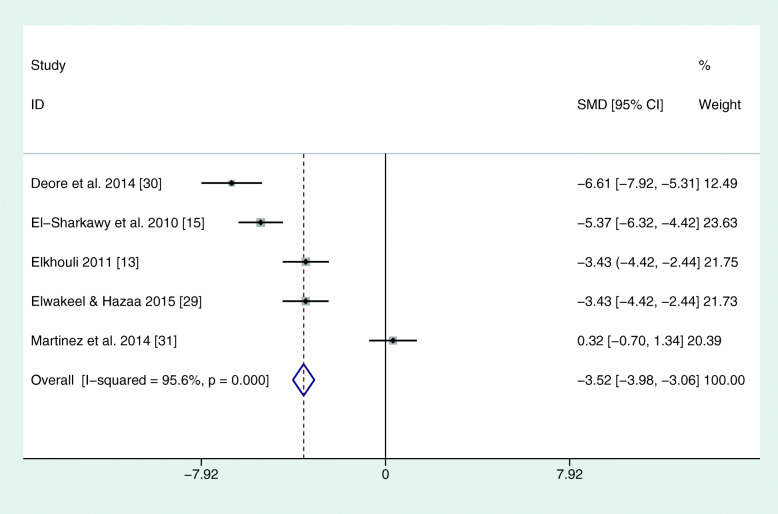
Meta-analysis of CAL after 3 months. SMD = standardized mean differences

**Fig. 4:**
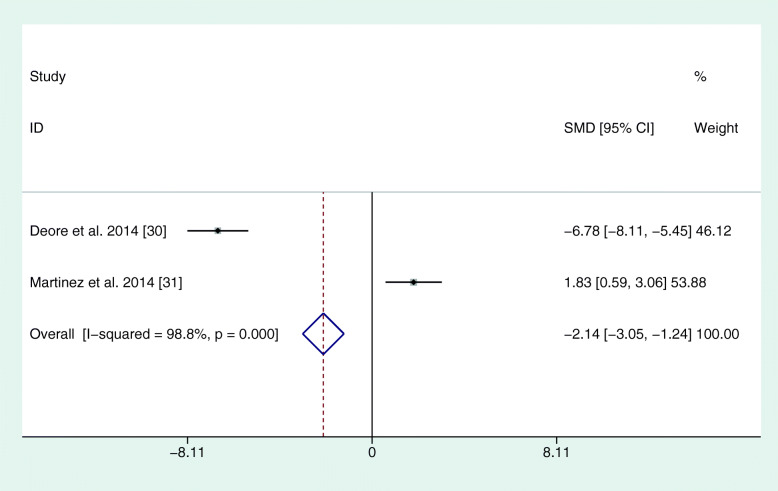
Meta-analysis of PD after 3 months for studies with omega-3 alone. SMD = standardized mean differences

**Fig. 5:**
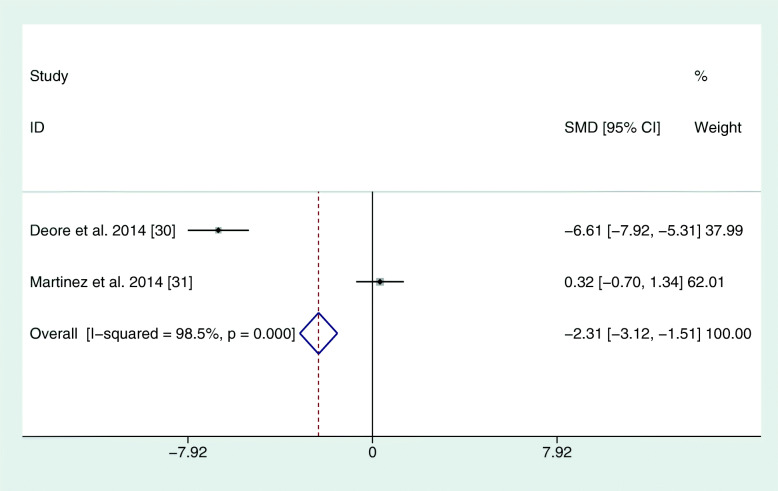
Meta-analysis of PD after 3 months for studies with omega-3 alone. SMD = standardized mean differences

The reviewed studies used different parameters to assess the periodontal condition and changes. In each study, PI, PD, and CAL were recorded. However, BOP at different time points was only found in three studies (El-Sharkawy et al., 2010; Keskiner et al., 2017; Martinez et al., 2014). In four studies, clinical parameters were always recorded by the same practitioner (see Table 2). In one study, different calibrated examiners were used at different time points or for different groups (El-Sharkawy et al., 2010). In this study, the reproducibility among examiners was 85%. Martinez et al. (2014) stated that several clinicians collected the clinical periodontal parameters, but no statement was made on the number of examiners. The reproducibility between the examiners in this case was 98% for PD and CAL. For the meta-analysis, all corresponding authors were contacted via e-mail for mean differences and standard errors. Only one author could deliver corresponding values as a full set of data (El-Sharkawy et al., 2010). Due to missing data, the meta-analysis was based on 3 months mean values. The study by Keskiner et al. was the only one that could not be included in the meta-analysis because it showed median values only, with no information on standard error. Hence, a meta-analysis of five studies revealed a positive effect for omega-3 on PD and CAL (see Figs. 2 and 3). There were no sufficient data for BOP. Furthermore, another meta-analysis was performed for two out of three studies that administered only omega-3 fatty acids without additional aspirin. Here, the study by Keskiner et al. could again not be included because of missing information on standard error. The meta-analysis of the two articles confirmed a positive effect of adjunctive omega-3 fatty acids on PD and CAL after 3 months (Figs. 4 and 5).

### Comparison of the results

By comparing the results of the six clinical studies, the adjunctive use of omega-3 fatty acids was able to significantly reduce PD in the experimental groups compared to those in the placebo groups in four of six studies (Table 3). Only one study showed an additional significant reduction of BOP in the test group compared to the control group (Deore et al., 2014). Significant reductions in the GI were observed in the studies by Elwakeel and Hazaa (2015), Deore et al. (2014), and Elkhouli (2011). When considering the PI, no changes were observed in three of six studies (Deore et al. [2014], El-Sharkawy et al. [2010], Keskiner et al. [2017]). Only the studies by Elwakeel and Hazaa (2015), Elkhouli (2011), and Martinez et al. (2014) confirmed that the PI also showed a significant reduction in the experimental groups. Regarding the CAL, four of six studies showed a significant decrease in the groups receiving adjunctive omega-3 fatty acids compared to the control groups (Elwakeel and Hazaa [2015], Deore et al. [2014], Elkhouli [2011], El-Sharkawy et al. [2010], and Martinez et al. [2014]). The studies by Martinez et al. (2014) and Keskiner et al. (2017) did not show this effect.

**Table 3:** Comparison of the results of the studies. The results of the measured clinical periodontal parameters in the test groups after administration of omega-3 fatty acids are compared with the placebo groups in the respective studies. PD = probing depth, BOP = bleeding on probing, GI = gingival index, PI = plaque index, CAL = clinical attachment level, ↑ = statistically significant increase in the test group compared to the control group, ↓ = statistically significant reduction in the test group compared to the control group, → = no change,, x = values were not determined here

Clinical periodontal parameter	Elwakeel and Hazaa (2015) [29]	Deore et al. (2014) [30]	Elkhouli et al. (2011) [13]	El-Sharkawy et al. (2010) [15]	Martinez et al. (2014) [31]	Keskiner et al. 2017 [32]
PD	**↓**	**↓**	**↓**	**↓**	→	→
BOP	x	x	x	→	→	→
GI	**↓**	**↓**	**↓**	↓	x	→
PI	**↓**	→	**↓**	→	→	→
CAL	**↓**	**↓**	**↓**	**↓**	→	→

### Risk of bias

Table 4 shows the juxtaposition of the risk of bias analysis, with a focus on general bias, omega-3 bias and periodontal therapy-related bias. The summary analysis (proportion) of the bias risk showed that, with a distribution of 83–95%, a high fulfillment of the quality criteria was provided.

**Table 4:** Risk of bias. According to Chee et al. (2016) [7] and the Cochrane Handbook by Schmucker et al. (2017) [18] Cochrane Germany

	Elwakeel and Hazaa (2015) [29]	Deore et al. (2014) [30]	Elkhouli (2011) [13]	El-Sharkawy et al. (2010) [31]	Martinez et al. (2014) [31]	Keskiner et al. (2017) [32]
General bias						
study design	1	1	1	1	1	1
randomisation	1	1	1	1	1	1
blinding	1	1	1	1	1	1
clear definition of inclusion and exclusion criteria	1	0	1	1	1	1
number of study participants	1	1	1	1	1	1
number of participants per study group	1	1	0	1	1	1
clear definition of the experimental groups	1	1	1	1	1	1
drop-out	1	1	1	1	1	1
control of the compliance of the participants	1	1	1	1	1	1
presentation of significant results	1	1	1	1	1	1
Omega-3-bias						
clear details of the medication and composition	1	1	1	1	1	1
clear indication of dosage	1	1	1	1	1	1
clear indication of the duration of the medication	1	1	1	0	1	1
clear indication of placebo (composition)	1	1	0	0	0	0
Periodontitis bias						
periodontal disease	1	1	1	1	1	1
selection of patients	1	0	1	1	1	1
treatment	1	1	1	1	1	1
periodontal parameters / indices	1	1	1	1	1	1
number of examiners	1	1	N/A	0	0	1
calibration of the examiner	N/A	N/A	N/A	1	1	1
time of recording of periodontal parameters	1	1	1	1	1	1
systemic diseases	1	1	1	1	1	1
smoker	1	1	1	1	1	1
Proportion	95%	87%	83%	87%	87%	95%

1 = quality criteria fulfilled, 0 = not fulfilled, N/A = no information

**Table 5:** Grading of Recommendations Assessment, Development and Evaluation (GRADE) according to Schünemann 2009 [20]

Quality assessment							Summary of findings				Importance
							No of patients		Effect	Quality	
No of studies	Design (Randomized controlled trial)	Limitations	Inconsistency	Indirectness	Imprecision	Other considerations	Omega 3	Control	SMD [95% Conf. Interval]		
PD after 3 months											
5	Yes	no serious limitations	no serious inconsistency^a^	no indirectness	serious imprecision	none	132	133	−3.931 [−4.446,-3.417]	moderate	high
CAL after 3 months											
5	Yes	no serious limitations	no serious inconsistency^a^	no indirectness	serious imprecision	none	132	133	−3.521 [−3.982,-3.059]	moderate	high
BOP after 3 months											
1	Yes	no serious limitations	no serious inconsistency	no indirectness	very serious imprecision	none	40	40	n/a	low	low

^a^the missing possible positive effect in reduction of PD and CAL could be due to low number of participants in Martinez et al. 2014 [31] and low-dose application of omega-3 in Keskiner et al. 2017 [32], *SMD* Standardized mean difference

### General bias

All studies showed a clear fulfillment of the quality criteria in terms of study design, randomization, and blinding (Table 4). A clear definition of the exclusion criteria was missing in the study by Deore et al. (2014). Here, only the inclusion criteria were mentioned. The number of study participants was clearly described in all studies, with no information on the number of participants per study group in the study by Elkhouli (2011). The exact distinction between the participating experimental groups was described in each study, and the dropout rate and reasons were mentioned. Whether the patients also demonstrated the desired compliance over the period of the studies was controlled in each study.

### Omega-3 bias

In all studies, clear statements were made on what the substituted omega fatty acids were composed of and in what dose they were administered (Table 4). No clear indication on the duration of the medication could be found in the study by El-Sharkawy et al. (2010). No information was provided on the composition of the placebo in the studies by El-Sharkawy et al. (2010), Elkhouli (2011), and Martinez et al. (2014).

### Periodontal therapy-related bias

The comparison of periodontal therapy-related bias showed that all studies except the one by Deore et al. (2014) provided a clear description of the included periodontal diseases (Table 4). Clear statements were made in all studies with regard to the selected treatment and periodontal parameters and indices used. Based on the number of investigators involved, the studies by Deore et al. (2014) and Elwakeel and Hazaa (2015) met the quality criteria of the bias analysis, whereas, in the study by Elkhouli (2011), no information was provided. In the studies by El-Sharkawy et al. (2010) and Martinez et al. (2014), there were several calibrated examiners, but their number was not mentioned. All studies met the criteria for describing the timing of the collection of periodontal parameters, presence of systemic diseases, and participation of smokers. However, the study by Elkhouli (2011) described that individuals who smoked ≤10 cigarettes per day were able to participate but lacked information about whether and how many smokers actually participated and which group they belonged to.

**Grading of Recommendations Assessment, Development and Evaluation (GRADE)** (Table 5).

Quality assessment according to Schünemann (2009) [20] did not show any serious limitations, inconsistencies, or imprecision in the five studies that examined PD and CAL after 3 months. Quality was evaluated as moderate for PD and CAL, while the importance was analyzed as being high. For BOP, only one study showed comparable data (mean values) at baseline and after 3 months. This led to a low quality assessment and low importance.

## Discussion

The subject of this systematic review and meta-analysis was the investigation of the effect of omega-3 fatty acids as a supplement to periodontal therapy. Four of the six studies included in this review showed a significant improvement in periodontal clinical parameters (PD and CAL) when periodontal therapy was performed with adjuvant omega-3 fatty acids compared to a placebo. The two included studies that did not show this effect had several limitations. Since Keskiner et al. (2017) supplemented low-dose omega-3 fatty acids, their results may indicate a dose-response relationship as the authors stated in their discussion. However, the study showed that supplementation of low-dose omega-3 fatty acids led to improved salivary TNF-alpha levels but might have been too subtle to lead to a clinical change. Likewise, analyzing the study of Martinez et al. (2014), a significant effect of omega-3 fatty acids on clinical parameters was not shown. However, here the small sample size (*n* = 7, test group; *n* = 8, control group) must be considered compared to the other studies included in this review. However, apart from the clinical parameters, in the test group serum, the EPA levels were significantly higher, and the AA/EPA ratio decreased at 4 and 12 months compared to those in the placebo group. Within the described limitations of the two studies mentioned above, the changes in saliva and serum might be seen as a precursor of a clinically measurable positive effect.

The overall predominantly positive effect of omega-3 supplementation supports the idea of using host-modulating therapeutic approaches more intensively in the treatment of periodontitis to prevent progressive tissue destruction. A special influence can be observed in PD and CAL, where, in most cases, a significant reduction occurred. Within the limitations of the available data, the meta-analysis confirmed these findings. In a recent review by Azzi et al. (2018), the influence on the severity of periodontitis of either supplementation of omega-3 fatty acids or omega-3-rich diet was evaluated [33]. No positive effect on clinical parameters was found. However, the authors concluded that increased EPA and DHA levels in the plasma could lead to decreased progression of periodontitis. In contrast to our literature review, only three studies with supplementation of omega-3 fatty acids were included. Only one of these three studies included periodontal treatment in terms of subgingival instrumentation with an extremely low number of cases, as noted above. Therefore, this heterogeneity does not allow any conclusions to be made regarding the adjunctive use of omega-3 during periodontal treatment but complements the findings of this review regarding the influence on periodontitis progression. This might be of increased interest in situations where adequate periodontal treatment is not possible or for general health issues or conditions that promote periodontitis progression, such as pregnancy or rheumatoid arthritis [34, 35].

Therefore, supplementation of omega-3 fatty acids might be an easy way to improve treatment in the short and long term in patients with periodontitis. In addition, due to the increasing problem of antibiotic resistance, alternatives to the systematic administration of antibiotics within periodontal therapy are urgently needed. The intake of fish oil capsules during periodontal therapy is easy and not too expensive to generally include it in a regular regimen. It might be harder for patients to obtain the same amount of DHA and EPA only by changing the composition of their meals. The recommended daily dose for EPA and DHA is 500 mg per day for cardiovascularly healthy individuals and 1000 mg per day for existing cardiovascular diseases. In the studies of this review, the daily doses were between 50 mg and 3000 mg. However, the results of this systematic review indicate that even lower applied doses of 300 mg [30] showed a significant improvement in the periodontal parameters comparable to the study with the highest dose of 3000 mg [29]. By contrast, the application extremely low doses as presented in the study by Keskiner et al. (2017) (twice daily) did not show a significant effect on clinical parameters as discussed above. In addition to the dose, the duration of administration of omega-3 fatty acids could be an important factor. From a nutritional health perspective, two servings per week of fatty sea fish should be sufficient to reach a basic requirement of 300 mg EPA/DHA per day. Currently, there is an average consumption of approximately 200 mg EPA/DHA per day in Germany [14], which indicates the relevance of the topic. Higher daily amounts can only be achieved through a combination of balanced, fish-rich food and the consumption of fortified foods (functional food, yoghurt, margarine, and eggs) and dietary supplements [11].

Despite the anti-inflammatory properties of omega-3 fatty acids, only one study showed an additional significant reduction in BOP [30], which suggests other systemic modes of action. Since three studies showed a significant improvement in other periodontal parameters regardless of the PI, this supports the view of the hypothesis of host-mediated dysbiosis [3]. Woelber et al. also showed that a reduction in gingival and periodontal inflammation can be realized only by an oral-health-optimized diet without a change in oral hygiene habits [36]. Thus, the treatment of periodontal disease should increasingly focus on host -modulation since the cause of the destructive processes in the periodontium seems not attributable to the presence of pathogenic biofilm alone. Thus, therapeutic approaches focusing only on plaque reduction may require more treatment effort due to a “chronic lack” of resolution of inflammation. Above all, dentists also have a nutritional advisory role. This was also emphasized in the current consensus conferences of the European Federation of Periodontology [37, 38]. From a practitioner’s point of view, a blood test at the beginning of periodontal therapy may be especially suitable for the determination of individual fatty acid status. It may include, among others, the so-called “omega-3 index” [8, 9]. This index indicates the percentage of EPA and DHA in the total amount of fatty acids in the red blood cell membranes. This reflects the exact tissue concentration of omega-3 fatty acids. Lowering the omega-3 index value seems to be a lethal risk factor for coronary heart disease [39]. Additionally, the ratio of omega-3 to omega-6 fatty acids may determine whether or not to administer omega 3-fatty acids during periodontitis therapy. This would also include a reduction or restriction in pro-inflammatory omega-6 fatty acids [8, 36] .

The literature review showed that, in three of the six studies, aspirin was administered in combination with omega-3 fatty acids. Omega-3 fatty acids in combination with aspirin may enhance their anti-inflammatory effects by promoting the formation of more potent resolvins and protectins [13–15]. Accordingly, the significant reductions or improvements in clinical periodontal parameters cannot be attributed solely to the mode of action of omega-3 fatty acids in these studies. However, one of three other studies showed that even without the addition of aspirin, the periodontal inflammatory situation was improved. Here, another meta-analysis including two studies using solely omega-3 fatty acids could confirm a significant positive effect. However, due to the small number of available studies and small sample size, further clinical studies are needed to prove a clear effect of the adjunctive use of omega-3 fatty acids alone. One negative aspect to consider when using aspirin continuously is the risk of developing gastritis or gastric ulcers [40]. In this context, the effect of salicylate blood levels can also be achieved through a plant-based, salicylate-rich diet (by spices and herbs such as chili, peppers, turmeric, cumin, or meadowsweet naturally contain a high concentration of salicylates) [41]. Future studies on omega-3 fatty acid supplementation with or without the combination of aspirin should also consider these possible dietary components.

In two of the analyzed studies, information was provided regarding the placebo used. In other studies, the composition was not mentioned. The compositions of placebos must be specified in the studies; otherwise, their pro-inflammatory properties and influences cannot be taken into account. Owing to the recognizable significant improvements in the periodontal parameters with adjuvant administration of omega-3 fatty acids in periodontitis therapy, a rethinking of the treatment concept is increasingly required in the direction of host-modulating therapy and in the sense of the hypothesis of host-modulating dysbiosis.

## Conclusions

The literature review and meta-analysis showed that omega-3 fatty acids as an adjunct to periodontal therapy showed significant benefits with regard to pocket depth reduction and attachment gain.

## Data Availability

All data generated or analyzed during this study are included I this published article.

## Abbreviations

*PICO*: Patient, intervention, comparison, outcome
*RCT*: Randomized clinical trial
*CAL*: Clinical attachment level
*PD*: Probing depth
*GI*: Gingival index
*BOP*: Bleeding on probing
*PI*: Plaque index
